# Dry Rolling/Sliding Wear of Bainitic Rail Steels under Different Contact Stresses and Slip Ratios

**DOI:** 10.3390/ma13204678

**Published:** 2020-10-20

**Authors:** Jiapeng Liu, Yingqi Li, Yinhua Zhang, Yue Hu, Lubing Shi, Haohao Ding, Wenjian Wang, Fengshou Liu, Shaobo Zhou, Tong Shi

**Affiliations:** 1 Metals and Chemistry Research Institute, China Academy of Railway Sciences, Beijing 100081, China; lyq_jh@163.com (Y.L.); nup@sina.com (Y.Z.); harve78@163.com (F.L.); hitshaoer@163.com (S.Z.); shitongthu@163.com (T.S.); 2 Tribology Research Institute, State Key Laboratory of Traction Power, Southwest Jiaotong University, Chengdu 610031, China; huyue0083@gmail.com (Y.H.); shilubing91@163.com (L.S.); dinghaohao520@163.com (H.D.); wwj527@swjtu.cn (W.W.)

**Keywords:** bainitic rail steel, contact stress, slip ratio, wear mechanism, friction coefficient, retained austenite

## Abstract

This study aims to deeply understand the effect of contact stress and slip ratio on wear performances of bainitic rail steels. The results showed that the wear loss increased as the contact stress and slip ratio increased. Based on the surface damage morphology and microstructural analyses, it revealed that the rolling contact fatigue wear mechanism played a significant role under the low slip ratio, but the dominant wear mechanism transferred to the abrasive wear at the high slip ratio. Meanwhile, the bainitic steel specifically presented worse wear resistance under the abrasive wear mode. Compared with the influence of a slip ratio, the increase in contact stress led to severer plastic flows and contributed to the propagation of cracks. In addition, the contact stress and slip ratio had the opposite effect on the friction coefficient, that is, the friction coefficient of bainitic steels behaved the inverse proportion with the contact stress, but positive proportion with the slip ratio. At last, the increase in slip ratio had more significant effect on the reduction of retained austenite (RA) than the enlargement of contact stress due to the fact that the RA would probably be removed before the martensitic transformation occurred under the abrasive wear mechanism.

## 1. Introduction

Pearlitic steel has been widely used in railway system, but the conventional pearlitic steel is susceptible to the formation of various defects, such as head checks and corrugation under actual rail-wheel contact conditions, which may lead to rail fracture and consume a great deal of maintenance cost and time [1,2,3]. Compared with traditional pearlitic steel, even though the welding of bainitic rail steel may lead to the appearance of high-carbon martensite, which is bad for the quality of welded joint, the technique of post-weld heat treatment has been conducted to solve this problem. Without regard to the welding limit, continuously cooled carbide-free bainitic steel is a kind of potential candidate for railway applications owing to its higher fracture toughness and fatigue resistance [2,3,4]. In addition, it has been reported that the thickness of bainitic lath can achieve to the nanometer level by the isothermal heat treatment [4,5,6]. These bainitic steels perform better mechanical properties than continuous cooling bainitic steels. However, the isothermal treatment is difficult to apply in the actual production line, especially in the production of 100-m-rail. Therefore, in our study, we focus on the continuous cooling carbide-free bainitic rail which was produced from the real rail manufacture.

In recent years, many field and laboratory studies have been carried out to investigate the wear performances and damage behaviors of pearlitic rail and wheel steels [7,8,9,10,11,12,13]. Ding et al. [9] compared the wear loss and surface damage of pearlitic wheel steels under different rotational speeds. The results showed that the wear loss of the pearlitic wheel steel increased, while the wear loss of the pearlitic rail steel declined with increasing the rotational speed. The surface morphology of the pearlitic rail steel turned from peeling to spalling at high speed. Huang et al. [10] considered that increasing normal force led to a rise in wear rate for pearlitic rail and wheel steels, and the crack angle became larger when the vertical force decreased. Guo et al. [11] reported that with the increase in slip ratio and contact pressure, the surface damage of pearlitic wheel steel transformed from pitting to delamination under the dry condition. Field investigations on microstructural evolution at early tonnage accumulation were conducted by Benoît et al. [13]. They proposed that cracks mostly propagated along the shear strained proeutectoid ferrite. Overall, the factors influencing the wear and the rolling contact fatigue (RCF) performance of pearlite steel are relatively clear.

As for the bainitic steel, several studies mainly focused on the comparison of the wear resistance [14,15,16,17,18,19] and RCF [3,15,16,20] between bainitic steels and pearlitic steels under the same working condition. However, the results were very contradictory. Some researchers believed that the improving wear resistance of bainitic steels was due to the surface microstructural transformation during wear process [19]. The transformation induced plasticity (TRIP) effect and the larger tolerance of degree of plastic strain [17,21] contributed to the increase in wear resistance. Some of these studies suggested that bainitic steels behaved better RCF resistance [15,16,20], which was due to the absence of surface and near surface microcracks [16] as well as the high fatigue strength [20].

On the other hand, in some previous studies, bainitic steels often presented poor wear resistance [22,23,24]. Some researchers proposed that the wear resistance of bainitic steels depended on the microstructure and thus on its transformation process [5,25,26]. It was also found that bainitic steels cannot obtain expected wear resistance due to its worse work hardening ability than pearlitic steels [15,16,18]. The controversial phenomenon was mainly due to the fact that too many diversified types of bainitic and pearlitic steels were used. These studied bainitic steels included continuing cooled bainite, isothermal bainite, real-field bainitic rail, small-ingot lab-made bainite, presenting various microstructure and mechanical properties. Meanwhile, the ultimate tensile strength of compared pearlitic steels varied from 880 MPa to 1380 MPa, the tensile strength grade of pearlitic steels certainly affected the comparison results. 

There have been relatively few studies concerning the intrinsic wear performances of bainitic steels under different working conditions, except in these contrastive researches between bainitic and pearlitic steels. Specifically, in practical wheel-rail contact, the contact stress and slip ratio should be considered as important parameters affecting the durability and tractive stability of wheel and rail system. It is still unclear how these two parameters affect the properties of bainitic steels. Studying the defect types and wear mechanisms of bainitic steels under different contact stresses and slip ratios could contribute to evaluate and predict the wear behavior and service life of bainitic rails. The results could also benefit the proper application of bainitic rail steel in complicated railway fields.

Within the complex microstructure of bainitic steel, retained austenite (RA) played an important role in the wear resistance [27,28,29] and fatigue resistance [2,25,30]. Liu et al. [30] considered that bainitic steels had excellent properties by blunting the crack tip on account of the presence of RA. While, some researchers believed that the wear resistance of bainitic steels was determined by the content of RA [5,30], other studies suggested that the stability of RA indeed played a significant role on promoting the damage resistance [2] and wear resistance [6,27,28] due to the TRIP effect. However, it is still elusive how the contact stress and slip ratio affect the transformation of RA in the bainitic steels. Therefore, in the present work, these scientific issues will also be investigated.

In this study, the effect of contact parameters on the wear performance of carbide-free bainitic rail steel was studied by changing the contact stress and slip ratio, in order to deepen the understanding of wear mechanism of bainitic steel under different working conditions. The findings would contribute to a better field application of bainitic rails in future.

## 2. Experiments

### 2.1. Experimental Materials and Parameters

In this experiment, MJP-30A RCF testing apparatus was used to conduct the dry rolling-sliding wear tests in order to evaluate the wear performance of bainitic rail steel. This test rig was assembled by Tribology Research Institute, State Key Laboratory of Traction Power, Southwest Jiaotong University. The rail and the wheel rollers were clamped on two rotating shafts driven by independent motors. The slip ratio can be adjusted by setting different rotating frequency of two motor shafts. The detail of sampling diagram can be found in the Ref. [28]. The speed of 500 rolling per minute (rpm) was applied in this experiment. The normal force was applied to the contact discs by a kind of hydraulic device. The friction torque and rolling cycles of the roller were measured by the torque sensor and revolution sensor, respectively. Once the total number of cycles for each group reached 50,000, the test would be terminated and the samples were disassembled from the tester for further subsequent measurements. Before, and after, the wear tests, the rail and wheel samples were cleaned using an ultrasonic cleaner and dried by a blower. Then rollers were weighted using an electron analysis balance. In order to diminish the experimental error generated during the experiment, each group of wear test was repeated twice. When the test deviations were too large, an additional repeated experiment would be performed.

**Figure 1:**
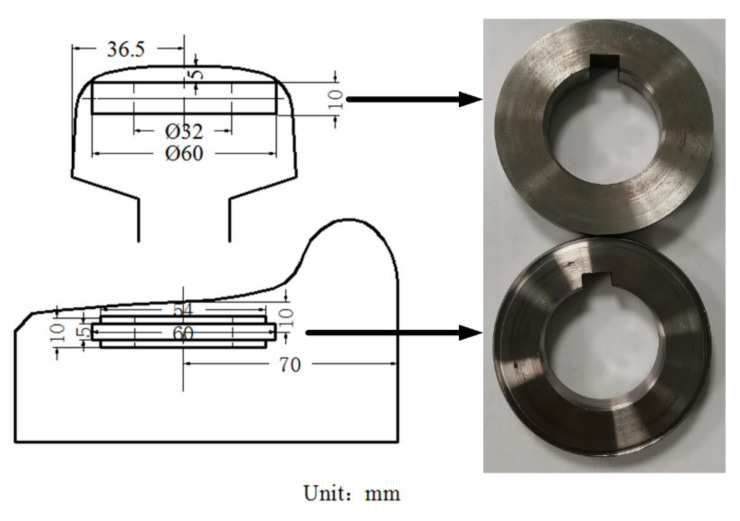
The left picture shows the sampling position and size on the real engineering components, the right picture shows the actual test rollers.

The bainitic rail steel (U22SiMn) and pearlitic wheel steel (CL60) were selected for wear tests. The bainitic rails were produced with the following procedures. The ingot of bainitic steel was heated to 1150 °C and held for 1 h. The initial hot rolling temperature began at 1150 °C, the finishing rolling temperature was not lower than 950 °C. At last, the air-cooled bainitic rail steel was reheated to 350 °C for two hours tempering. The temperature of 350 °C could not lead to the temper brittleness based on the previous experimental results. The chemical composition and mechanical properties are shown in Table 1 and Table 2. Figure 1 schematically shows the sampling position and size for respective rail and wheel rollers. The wheel and rail rollers were cut off from the wheel tread and the as-rolled rail head. The diameter of roller was designed as 60 mm and the contact width was 5 mm.

**Figure 2:**
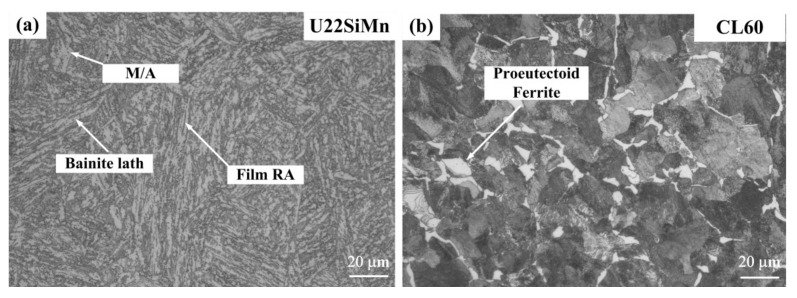
OM observation of the microstructures of bainitic steel; and (**a**) pearlitic steel (**b**).

Figure 2 shows the optical microscope (OM) images of the microstructures of bainitic rail and pearlitic wheel. The original microstructure of the bainitic steel is more complicated than the pearlitic steel, consisting of bainitic ferrite (BF) lath, small amounts of film RA, blocky M/A and twin-morphology martensite. CL60 wheel steel is composed of the proeutectoid ferrite and lamellar pearlite structure. The thickness of bainitic lath in base metal is about 690 nm, and the lamellar spacing of pearlite is close to146 nm. More information on the microstructure of specimens can be found in the Ref. [18,31].

In this experiment, Hertzian contact criterion [32] was adopted to simulate the cylinder on cylinder contact condition, so that the maximum Hertzian contact stress at the contact zone could be consistent with the actual field condition. However, the contact zone in our case was about 3.5 mm^2^ which was not close to the elliptical contact patch in the real wheel-rail contact which is about 110 mm^2^. The value of Young’s modulus selected in this calculation was 205 GPa, and the value of Poisson’s ratio was 0.3. The applied normal forces were designed to 4300 N, 2850 N and 500 N. According to the Formulas (1) and (2) [33] the contact stresses were 1430 MPa, 1150 MPa and 500 MPa, respectively. The parameters of different testing groups are shown in Table 3,

(1) b=2F1−v12E1+1−v22E2𝜋l1d1+1d2

where *b*: Contact half-width; *F*: Applied normal force; *ν*1, *ν*2: Sample-1(2) Poisson’s ratio; *E*1, *E*2: Sample-1(2) elastic modulus; *d*1, *d*2: Sample-1(2) diameter; l: Contact length of cylinders.

The maximum contact stress is:

(2) $$p_{max}=\frac{2F}{\pi bl}$$

### 2.2. Characterizations

An electronic analytical balance (JA4103, China) with an accuracy of 0.0001 g was used to measure the weights of the rail roller and wheel roller before and after the experiment. Each sample was weighed three times and the average value obtained. The micro-hardness of the surface and cross-section of test specimens was measured by the Vickers hardness tester (MVH-21, Japan). A roughness meter (Jb-5c, China) was used to record the surface roughness of the worn samples. Longitudinal cross-sections (with the arc length of about 8 mm) of the rail/wheel discs were cut off along the rolling direction for metallographic analysis. Each cross-sectional specimen was inlaid in the resin to protect the edge of the worn surface. Ground with SiC paper of 500 and 1000 grit, polished with 3.2 μm diamond, and etched with 4% Nital for 10 s, the microstructure of each section could be clearly analyzed with the optical microscopy (OM,DMI5000 M, Leica, Japan) and scanning electron microscopy (SEM,Quanta 400, FEI, America).

The volume fraction of retained austenite was determined by X-ray diffractometry (Bruker D8, Germany) which was equipped with Co-Kα radiation. The information of diffraction X-ray in the range of 30–115° was collected by the Lynxeye XE detector in scanning step of 0.02°. The calculations of the volume fraction of RA were based on the integrated intensity of (200)_α_, (211)_α_, (200)_γ_, (220)_γ_ and (311)_γ_ diffraction peaks, the deviation value in retained austenite fraction is about 1.0%. The relevant method of estimation and equations can be seen in the paper written by Xu [34].

## 3. Results

### 3.1. Wear Behaviors

**Figure 3:**
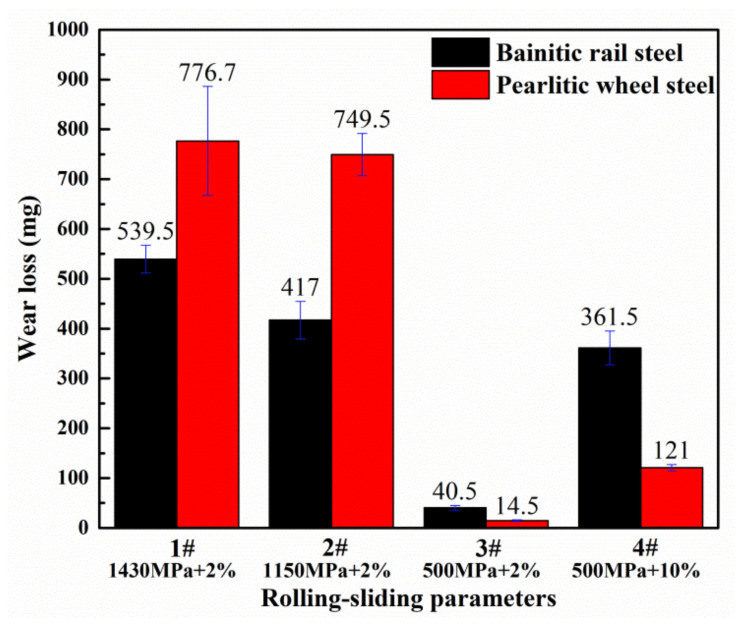
The wear loss of bainitic rail and pearlitic wheel rollers.

The mass loss of the wheel and rail samples for four testing sets was calculated and shown in Figure 3. It can be seen the wear loss of the bainitic rollers is about 30–44% less than that of pearlite wheel rollers in 1# and 2#. It suggests that the wear resistance of bainitic steel is superior to the pearlitic CL60 steel under large contact stress level. It should be noted that, with an increase of slip ratio (4#), the wear loss of the bainitic rail steel and CL60 wheel steel present much more increase than the 3# specimens at low slip ratio.

**Figure 4:**
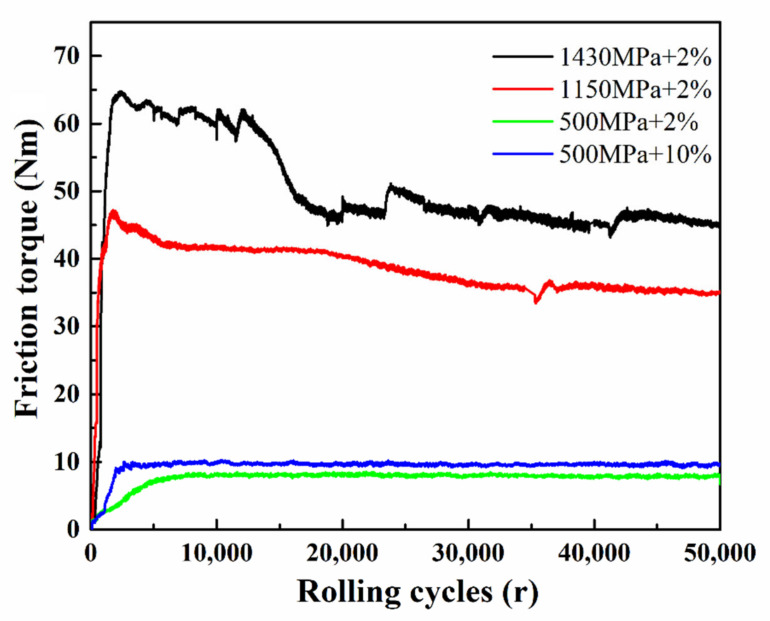
The evolution of real-time friction torques during four sets of tests.

The friction coefficient of the wheel-rail contact zone is an important factor affecting wheel-rail wear and traction in actual conditions. Therefore, in present work, the coefficients of friction under different contact stresses and slip ratios were specifically investigated. The real-time friction torque could be recorded by the MJP-30A RCF testing apparatus as shown in Figure 4. It can be seen that there was an obvious running-in period in the test of high contact stress (1# and 2#), where the values of friction torques were large and instable. Then, the wear state gradually transferred from the running-in period to the stable status, where the friction torque turned to be stable after 15 k rolling cycles. The friction torque was defined as M = T_f_ × R, where T_f_ represented the friction force and R was the radius of the specimen. At the end of the tests, the corresponding friction torque and roller radius for each test group would be recorded and measured. Finally, the friction force and the coefficient of friction at stable wear condition (50 k rolling cycles) can be obtained in Table 4.

### 3.2. Investigations on Worn Surface

**Figure 5:**
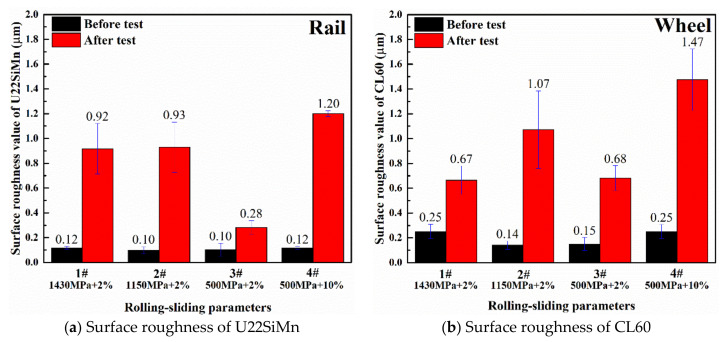
The roughness values of bainitic steel and CL60 wheel before and after wear tests.

The surface roughness may affect the coefficient of friction between the discs and the distribution of stresses near the surface during test [35]. Pin-on-disc tests were also performed by the researchers [36] who discovered that the roughness value produced an effect on the coefficient of friction in dry and lubricated sliding. Therefore, in order to minimize the effect of initial surface roughness on tests, the experimental specimens were managed to approximately behave the same surface roughness (0.1 μm for bainitic rollers). The roughness values of rail and wheel samples before and after tests are shown in Figure 5. It can be seen that the worn surface roughness of 3# bainitic rail was the lowest (about 0.28 μm), but the 4# bainitic rail showed the highest roughness (about 1.2 μm) due to high slip ratio. The surface roughness (about 0.92 μm) of bainitic rail steels under larger contact stress (1# and 2#) was not significantly different from each other after tests, while their roughness values were much higher than that in test 3#.

**Figure 6:**
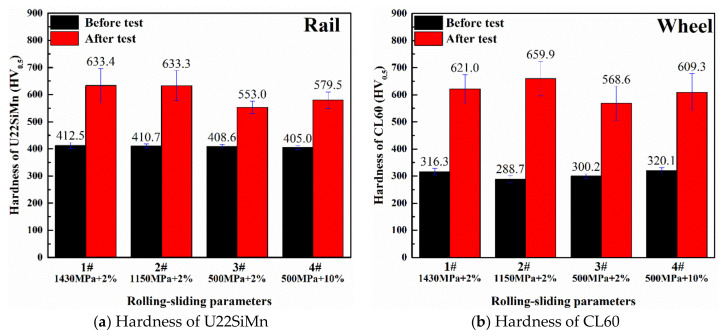
Surface hardness of rail and wheel specimens.

After running 50,000 cycles, the post surface hardness was measured with the micro-hardness tester by averaging five points on the worn surface (as shown in Figure 6). It showed that the post surface hardness was prone to increase with the raise of contact stress. Compared with 3# and 4# specimens, it can be seen that the increase of slip ratio caused a bit of increase in surface hardness of bainitic and wheel rollers simultaneously.

It should be noted that the surface hardness of pearlitic wheel rollers was generally higher than that of bainitic rollers after the test, indicating relatively excellent work hardening capacity in pearlitic steels. The amount of strain hardening (i.e., the increase in hardness during sliding divided by initial hardness before test) [17] for bainitic rollers (1#–4# tests) was 0.53, 0.54, 0.35, and 0.43, respectively. However, for wheel rollers (1#–4# tests), the values were 0.96, 1.29, 0.89, and 0.90, respectively. It indicates that the amount of strain hardening tended to increase with the increase in contact stress and slip ratio. Obviously, the bainitic rollers demonstrated the lower amount of strain hardening than pearlitic wheel rollers, which was consistent with the opinion reported by Yokoyama et al. [20] that the amount of strain hardening decreased as initial hardness increased, regardless of the type of microstructures.

**Figure 7:**
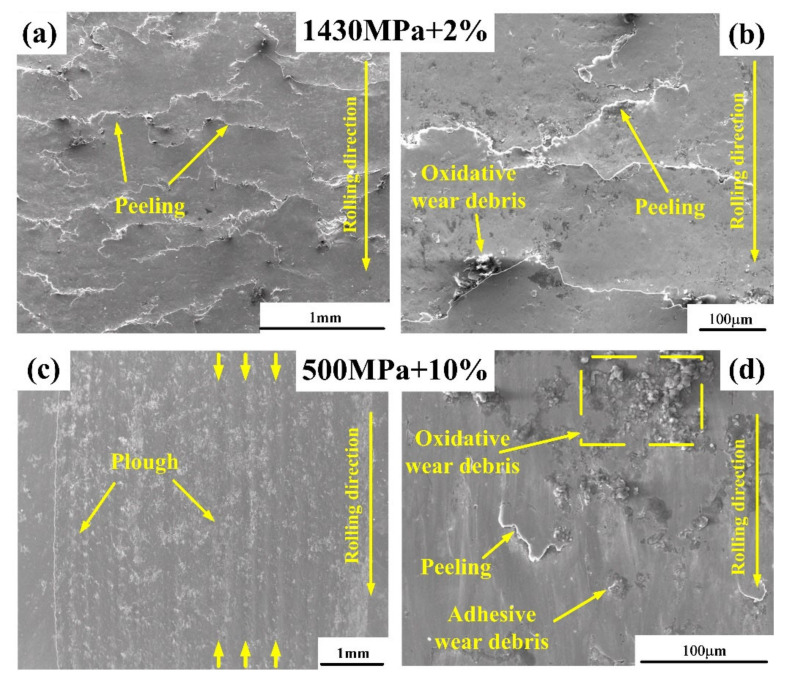
The surface damage morphologies of bainitic steel in SEM after tests under different wear parameters: (**a**,**b**) 1430 MPa + 2%; (**c**,**d**) 500 MPa + 10%.

In order to investigate the influence of contact stress and slip ratio on the surface damage of bainitic steels, scanning electron microscopic analyses were conducted (as shown in Figure 7). It can be obviously seen that the damage morphology of the worn surface in 1# was different from that in 4# specimen. As shown in Figure 7a,b severe peelings were produced on the worn surface under the condition of 1430 MPa + 2%. Moreover, under the function of cyclic loading, these cracks propagated and formed a few delaminations. However, in Figure 7c,d only slight cracks were observed. The amount of oxidative wear debris increased due to the higher temperature obtained at 10% slip ratio. Meanwhile, ploughing morphology with cutting traces can be observed on the worn surface under the condition of 500 MPa + 10%, which was not found in other three groups with low slip ratio of 2%.

These morphological features indicated that different wear mechanisms existed in bainite steels under different wear conditions. The surface morphology with pitting and fatigue cracks (1#) indicated that there was strong surface fatigue wear with severe plastic deformation. The formation of furrow morphology was due to the cutting of materials by the hard particles which slid between the contact surfaces. It can be seen from the Figure 7d that these hard particles should be the wear debris attached on the surface. It demonstrated that the abrasive wear had been mainly worked on the 4# bainitic roller.

### 3.3. The Analysis of Cross-Sectional Microstructure

**Figure 8:**
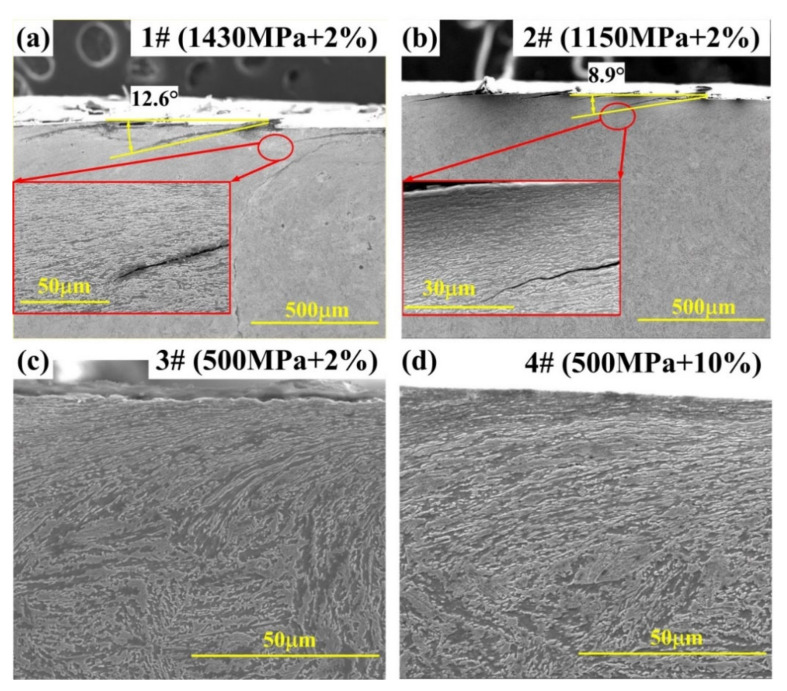
SEM images showing the cross-sectional microstructure of bainitic rail steels (**a**) 1430 MPa + 2%, (**b**) 1150 MPa + 2%, (**c**) 500 MPa + 2% and (**d**) 500 MPa + 10%.

For studying the development of cracks, cross-sectional microstructures of bainitic steels were analyzed. The number of cracks, crack depth, and inclination angle were measured from the SEM images (as shown in Figure 8) and listed in the Table 5. The SEM images clearly showed that some severe plastic flows were generated in the subsurface layer during the wear process, and the microstructure near the surface was significantly aligned with the wear direction. 

Under the stress of 500 MPa (3#, 4#), cracks were almost invisible in the bainitic rail surface, but there were some evident cracks on the worn surface in the cases of 1430 MPa (1#) and 1150 MPa (2#) (shown in Table 5). Moreover, the average inclination angle of cracks was increasing with the enhancement of contact force and slip ratio. However, the change in slip ratio did not significantly influence the initiation of cracks on the worn surface. It is noteworthy that, the increase in slip ratio led limit increase in average crack depth. These phenomena indicated that the formation and growth of RCF cracks were mainly affected by the contact stress.

**Figure 9:**
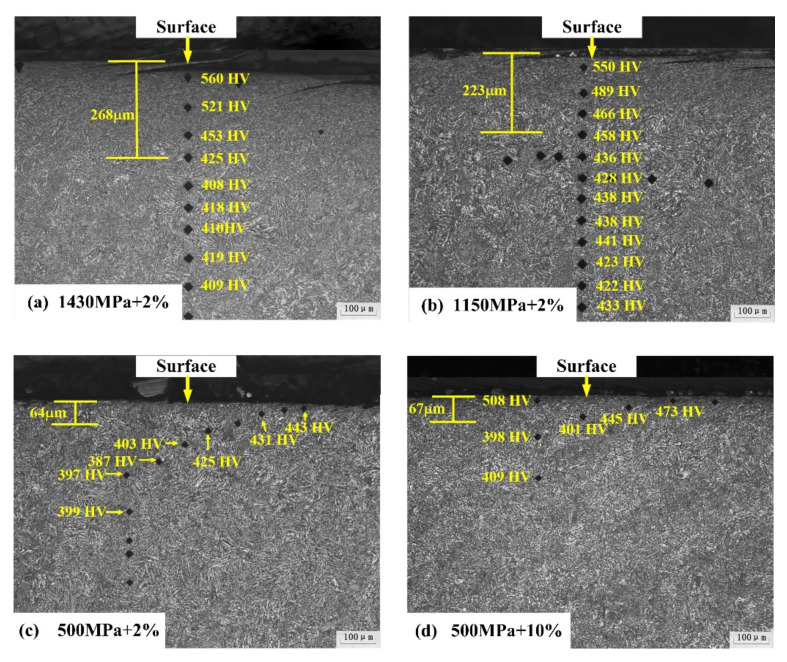
Microhardness of subsurface layer in the tested bainitic rail steels (HV 50 g) (**a**) 1430 MPa + 2%, (**b**) 1150 MPa + 2%, (**c**) 500 MPa + 2% and (**d**) 500 MPa + 10%.

Figure 9 showed the microhardness gradient and plastic deformation features of the subsurface layers corresponding to four kinds of wear conditions. Microhardness tester was applied to measure the hardness gradient from the surface to the matrix, with the parameters of 50 gf loading and 15 s duration time. It can be seen that the maximum hardness of the subsurface layer was about 560 HV in the 1# with the largest contact stress. Moreover, the surface hardness gradually increases with the increase in the slip ratio with a comparison of 3# and 4#.

Specimen in 1# test had the largest deformed layer with a thickness of about 268 μm, and the smallest plastic area with the depth of about 64 μm was observed in the subsurface layer of 3# specimen. Notably, with the same slip ratio, the depth of plastic deformation layer of bainitic rail steel increased with the increase of contact stress. However, in tests of 3# and 4#, the thickness of the plastic deformation layer was nearly similar. It revealed that the influence of slip ratio on the depth of plastic deformation layer was less than the contact stress. Meanwhile, according to Table 5 and Figure 9, these results revealed that the crack depth was positively proportional to the depth of the plastic layer.

### 3.4. The Transformation of Retained Austenite during Wear Tests

**Figure 10:**
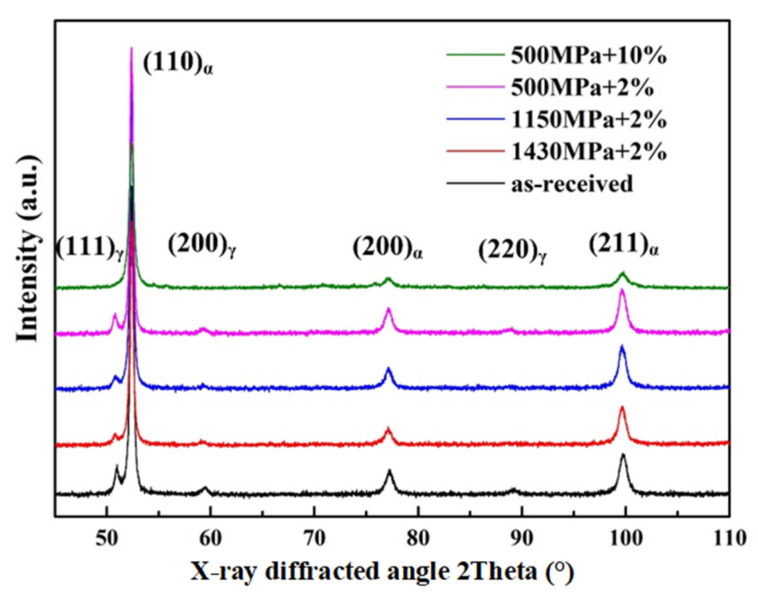
Comparison of XRD spectrums obtained from the worn surface with four kinds of wear conditions.

In the present test, X-ray diffraction experiments were performed on the contact surfaces before and after the test, as shown in Figure 10. The estimations of the volume fraction of RA were recorded in Table 6. The original volume fraction of RA was about 9.33%. After wear tests, the volume fractions of RA in the samples of 1#–4# were 4.77%, 4.96%, 8.98%, and 0.58%, respectively. It indicated that the RA content on the worn surface of the bainitic roller gradually decreased with the increase in the contact stress. However, in consideration of slip ratio, only 4% of the original RA was transformed in the experiment 3#, but 94% of the original RA was transformed in the experiment 4# with the same contact stress but different slip ratio. Therefore, it is obvious to see that both contact stress and slip ratio have influence on the transformation of RA in bainitic steel, but the effect of slip ratio on RA was more significant.

## 4. Discussion

### 4.1. Effect of the Contact Stress on the Wear Resistance and RCF Resistance of Bainitic Steel

**Figure 11:**
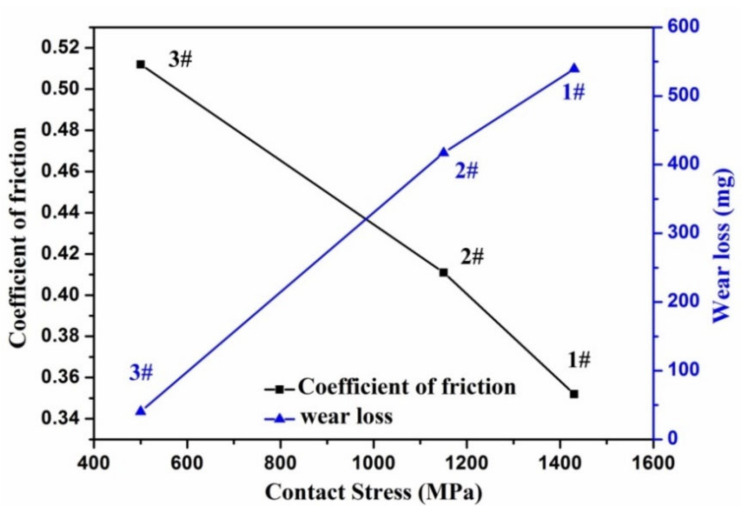
The relation among coefficient of friction, wear loss and contact stress in the samples of 1#, 2# and 3#.

Comparing three sets of experiments at 2% slip ratio, i.e., 1#, 2# and 3#, it can be seen that the wear loss nearly linearly increased with the contact stress as shown in Figure 10. Similarly, it is common to predict that the friction force should increase with the increase in the contact stress as shown in Table 4. However, from Figure 11, it is interesting to see that the coefficient of friction decreases with the rise of contact stress for the studied carbide-free bainitic rail steel at the same condition of slip ratio.

Comparison with the wear behaviors of pearlite rail steels in paper [37], the authors found that the increases in axle loads enlarged the friction coefficient significantly, and the values were about 0.36, 0.42, and 0.53 for the axle loads of 16, 21, and 25 t, respectively. However, based on the research of Chen et al. [38], the influence of the axle load on the adhesion of wheel and rail was not proportional, but the adhesion depended on the surface roughness and the running speed of a vehicle. Therefore, besides the influence of the contact pressure, rolling velocity and slip ratio, the friction coefficient could also be affected by the microstructural properties of materials. In the present study, it is proved that the bainitic steel behaves a lower friction coefficient at the large axle load, which may be opposite with the results in pearlitic steels [37]. The influence of microstructural features, such as work hardening behavior, surface roughness or elastic-plastic deformation behaviors, on the friction coefficient should be deeply studied in the future work. 

As shown in Figure 8 and Figure 9, after 50 k rolling cycles, micro-plastic flows and cracks were generated beneath the rolling contact surface. There was a significant difference in the extent of plastic flow beneath the rolling contact surface. The first set of tests with high contact stress wasidentified with the largest work hardening depth (268 μm). It reveals that the development of plastic flow increases with the rise of contact stress. Moreover, according to the statistic estimation of the depth and number of cracks (Table 5), it indicates the same trend with the development of plastic flow. This relevance fact could be explained by the shakedown theory [39,40]. The well-known shakedown diagram predicts the relationship between the friction coefficient and loading factor (contact stress (p_0_)/shear yield strength (k)). When the parameter of p_0_/k exceeds the prescribed shakedown limit of studied bainitic steel, there will be a larger possibility to form severe plastic flows and make the growth of cracks. The shear yield strength (k) could be calculated by the formula [1] as follow, k = 1.89*A*HV, where A stands for the yield-strength ratio (0.875 according to Table 2), and HV represents the hardness (Figure 6). In our case, the parameter of p_0_/k for these three sets of tests with the contact stress of 1430 MPa, 1150 MPa and 500 MPa can be calculated as 1.05, 0.85 and 0.42, respectively. Therefore, the larger contact stress has much more tendency to develop plastic flows and contribute to the propagation of cracks in the surface layer.

### 4.2. Effect of the Slip Ratio on the Wear Resistance and RCF Resistance of Bainitic Steel

With the same condition of applied contact stress (500 MPa), compared to wear tests of 3# and 4#, it can be seen that the wear loss of bainitic steel at 10% slip ratio was almost nine times of the wear loss at 2% slip ratio (as shown in Figure 3). Meanwhile, the friction coefficient at high slip ratio was greater than those at the low slip ratio (present in Table 4). It indicates that the increase in slip ratio plays a more significant role on aggravating wear loss than the enlargement of contact stress.

The reasons for the above results can be explained by two common wear mechanisms. Based on the surface observation in Figure 7, it displayed that a large amount of surface RCF cracks existed at the low slip ratio specimens (1#, 2# and 3#), but a few clear trace of furrows and stacked wear debris presented in the high slip ratio specimen (4#), indicating that the main wear mechanism in the low slip ratio tests was RCF wear, while the wear mechanism in the high slip ratio test was dominated by the abrasive wear.

**Figure 12:**
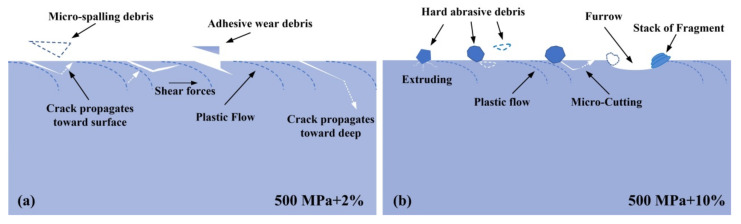
Wear mechanism maps at the condition of (**a**) 500 MPa + 2%, (**b**) 500 MPa + 10%.

Schematically shown in Figure 12a, for the 3# test with 500 MPa + 2%, surface cracks prefer to be generated and propagated along the plastic flow. A part of cracks would propagate toward subsurface, but a few cracks would connect with each other due to the effect of shear stress and thus form the micro-spalling debris. However, when the high slip ratio applied in the test environment, the initially produced micro-spalling and adhesive wear debris would, either extrude into the worn surface or invade along the open mouth of cracks, leading to a direct contact between the wear debris and potential peeling layers. Under the micro-cutting behavior [41], the formation of peeling debris would be accelerated more rapidly than the normal RCF progress. As a result, the wear rate of test 4# was much higher than that of 3# under the same contact stress, even approached to the wear rate at a higher contact stress level (test 2#).

In addition, from the analysis of surface roughness in the Figure 5, it should be noted that the surface roughness caused by the fluctuation of the groove and bulged rough peaks at 10% slip ratio is higher than other test samples featured with RCF cracks (including 1#, 2# and 3#), no matter suffered with large or small contact stress.

By comparing the crack features between 3# and 4# in the Table 5, it can be seen that the number and average depth of RCF cracks are not much different, revealing that the increase in slip ratio made less influence on the development of cracks than the enlargement of contact stress.

### 4.3. Influence of the Contact Stress and Slip Ratio on the Transformation of RA

Based on the XRD spectrums in Figure 10, it can be visually seen that the (111)γ peak almost disappeared after the wear test with 10% slip ratio. The volume fraction of RA in the worn surface of 4# sample reduced to 0.58%, which was much less than other three sets of specimens. Compared with the friction force between 1# and 4# (Table 4), it can be seen that the friction force of 4# (326 N) was far less than that in 1# (1512 N). It indicates that the friction-related tangential force is not a crucial factor for the trigger of martensitic transformation (MT) of RA. Meanwhile, it should be noted that the volume fraction of RA between 1# and 2# (Table 6) is nearly similar. This fact reveals that the increase in contact stress could facilitate the transformation of RA at low stress condition (500–1150 MPa), but confronts some limitation at high stress condition (1150–1430 MPa).

The effect of RA on the wear resistance of bainitic steel has been extensively studied. Some scholars believed that the RA on the worn surface underwent martensitic transformation, which resulted in a dramatic increase in hardness, compared with materials containing no RA [3,25,42]. Many publications [5,42,43] pointed out that the RA can promote wear resistance through the TRIP effect due to the increase in work hardening capacity of the material. However, in the present study, though the 4# bainitic roller suffered with the largest transformation of RA, the hardness of worn surface (579.5 HV) did not obviously exceed other sets of specimens (as shown in Figure 6), and also showing the worst wear resistance. Moreover, it has been well-known that the transformation of RA to martensite can induce internal compressive stress which would retard the propagation of RCF cracks [30]. However, according to the Table 5, there is no prominent advantage of reducing the number and depth of RCF cracks for 4# bainitic roller.

Therefore, it is reasonable to presume that the evolution of RA in the 4# specimen during the rolling/sliding test may not go through the MT. There is an interesting idea proposing that the stability of RA has a greater influence on the wear resistance than the amount of retained austenite [27,28]. On the one hand, when the RA featured with low stability, it would be too easy to transform resulting in little improvement of wear resistance. On the other hand, when the RA behaved with high stability, it would be too difficult to achieve the MT but undergo with other transitions, leading to small promotion of wear resistance. As for 4# sample applied with the low contact stress and high slip ratio, the temperature on the contact zone would be higher than other conditions with the low slip ratio [44]. The increase in temperature could contribute to the stabilization of RA. Meanwhile, the mechanical transformation of RA may be lack of driving force due to the low applied contact stress. Therefore, the RA in 4# bainitic steel may be difficult to achieve the MT under low contact stress and high slip ratio. The disappearance of RA could be related to the abrasive wear model at the high slip ratio, which would probably remove the RA phase before the MT occurred. 

In summary, the bainitic steel showed the better wear resistance at the low slip ratio due to its excellent toughness acting on the RCF wear mechanism. However, the bainitic steel is not suitable for the small-radius curves with high slip ratio due to its worse wear resistance under the abrasive wear mechanism.

## 5. Conclusions

1. The wear loss increases with the rise of the contact stress and slip ratio, but the slip ratio plays a more crucial role on aggravating wear loss than the contact stress.
2. The rolling contact fatigue wear mechanism plays a significant role under the low slip ratio condition, but the dominant wear mechanism transfers to the abrasive wear at the high slip ratio. The bainitic steel is not suitable for the small-radius curves with the high slip ratio due to its worse wear resistance under the abrasive wear mode.
3. The enhancement of contact stress has much more tendency to develop plastic flows and contribute to the propagation of cracks in the bainitic steels than the increase in slip ratio.
4. The friction coefficient of bainitic steels has an inverse proportion with the contact stress, but positive proportion with the slip ratio.
5. The volume fraction of RA decreases with the increase in contact stress and slip ratio. The increase in slip ratio has a more significant effect on the reduction of RA than the enlargement of contact stress due to the fact that the RA phase would probably be removed before the MT occurred under the abrasive wear mechanism.

## Figures and Tables

**Table 1:** Chemical composition (% of wt.) of selected rail and wheel steels.

Component	Steel Grade	C	Si	Mn	P	S	Cr + V
Rail	U22SiMn	0.20–0.23	1.30–1.35	2.00–2.10	≤0.025	≤0.025	≤0.589
Wheel	CL60	0.58–0.60	0.20–0.25	0.72–0.74	≤0.030	≤0.030	≤0.14

**Table 2:** Mechanical properties of selected rail and wheel steels.

Component	Steel Grade	R_m_/MPa	R_p0.2_/MPa	A/%	Bulk Hardness/HBW
Rail	U22SiMn	1283	1123	16.0	401
Wheel	CL60	932	593	17.0	286

**Table 3:** Summary of experimental parameters.

Testing Groups	Normal Load (N)	Contact Stress (MPa)	Slip Ratio (%)	Rolling Speed of Wheel (rpm)
1#	4300	1430	2	500
2#	2850	1150	2	500
3#	520	500	2	500
4#	520	500	10	500

**Table 4:** The friction-related parameters at 50 k rolling cycles for four sets of tests.

Test Group	Normal Load(N)	Friction Torque (Nm)	Disc Radius (mm)	Friction Force (N)	Coefficient of Friction
1#	4300	45.20	29.89	1512	0.352
2#	2850	35.00	29.93	1169	0.411
3#	520	7.96	29.93	266	0.512
4#	520	9.56	29.35	326	0.627

**Table 5:** Statistics of crack depth and angle.

Testing Groups	Number of Cracks	Average Depth (μm)	Max Depth (μm)	Average Inclination Angle (°)
1#	18	85 ± 4	126	9.1 ± 1.3
2#	17	38 ± 3	71	6.7 ± 1.0
3#	3	8 ± 2	11	6.6 ± 1.0
4#	4	11 ± 2	24	8.0 ± 1.5

**Table 6:** Volume fraction of RA in bainitic rail steels with four kinds of wear conditions (about 1.0% deviation value in retained austenite fraction).

	1# (1430 MPa + 2%)	2# (1150 MPa + 2%)	3# (500 MPa + 2%)	4# (500 MPa + 10%)
VF of RA before wear	9.33%	9.33%	9.33%	9.33%
VF of RA after wear	4.77%	4.96%	8.98%	0.58%
